# Developmental and Stress-Mediated Transcriptional Shifts in Riboflavin Metabolism Pathway in Arabidopsis

**DOI:** 10.3390/genes17010016

**Published:** 2025-12-25

**Authors:** Dikran Tsitsekian, Panagiota Mylona, Efstratios Kamargiakis, Stamatis Rigas, Gerasimos Daras

**Affiliations:** Department of Biotechnology, Agricultural University of Athens, 11855 Athens, Greece; dtsitsekian@aua.gr (D.T.); stud320080@aua.gr (P.M.); skamargiakis@aua.gr (E.K.); srigas@aua.gr (S.R.)

**Keywords:** Arabidopsis, riboflavin, flavins, vitamin B_2_, abiotic stress

## Abstract

**Background**: Flavin cofactors, flavin mononucleotide (FMN) and flavin adenine dinucleotide (FAD), are indispensable for plant metabolism, supporting photosynthesis, photorespiration, mitochondrial electron transport, nitrogen assimilation, and cellular redox balance. Both cofactors derive from riboflavin (vitamin B_2_), which plants synthesize de novo, unlike animals, which rely on dietary intake. While the riboflavin biosynthesis pathway has been biochemically well-characterized, its transcriptional regulation and cellular organization remain poorly understood. **Methods**: Here, using large-scale transcriptomic datasets as well as co-expression and cis-element analyses, we systematically investigated the expression dynamics of riboflavin metabolism genes in *Arabidopsis thaliana*. In addition, HPLC was employed to monitor flavin level fluctuations in plants under abiotic stresses. **Results**: Most genes displayed strong expression in photosynthetic and reproductive tissues, consistent with elevated metabolic demands for flavins in redox reactions and energy metabolism. Under osmotic stress, *RIBA1*, *RIBA3*, *PYRD*, *PYRR*, *COS1/LS,* and *RS*, genes encoding enzymes involved in the early and intermediate steps of riboflavin biosynthesis were transcriptionally downregulated. In contrast, *RIBA2*, *FHY1/PYRP1* and *FMN/FHY* were upregulated, whereas *FADS1* and *NUDX23*, genes encoding enzymes responsible for interconversion between FMN and FAD, were suppressed. Gene expression responses are consistent with the maintenance of flavin homeostasis, affecting flavin level changes under abiotic stress. **Conclusions**: This study establishes a comprehensive framework for the transcriptional regulation of flavin biosynthesis in plants. The findings reveal stress-responsive reprogramming of flavin metabolism and identify promising strategies for engineering crops for biofortification, metabolic efficiency, and stress resilience.

## 1. Introduction

The flavin cofactors flavin mononucleotide (FMN) and flavin adenine dinucleotide (FAD) are essential for numerous metabolic processes for all organisms. Photosynthesis, photorespiration, mitochondrial electron transport, nitrogen assimilation, and cellular redox homeostasis in plants depend on flavins [1]. The precursor of FMN and FAD is riboflavin (vitamin B_2_), which is synthesized in plants and microorganisms, whereas animals depend entirely on dietary intake to meet their demands. In plants, FMN and FAD cofactors are essential for growth under normal conditions and for various stress responses [2]. Although the riboflavin metabolic pathway has been largely elucidated at the biochemical level, there is a lack of comprehensive information regarding flavins transcriptional regulation. Understanding the spatiotemporal expression patterns of genes involved in riboflavin synthesis across developmental stages and in response to external stimuli is critical for understanding flavin metabolism control in the plant body. Moreover, information on the subcellular localization of flavins’ metabolic enzymes is crucial, especially for targeted approaches aiming to genetically modify the pathway for biotechnological applications and stimulate flavin coenzyme availability for metabolic engineering or enhancing plant stress resilience and adaptation. The post-genomic era resulted in advancements in high-throughput technologies, generating vast quantities of omics data across models and diverse plant species [3]. The availability of these datasets significantly enhances reproducibility and cross-study validation, making these datasets indispensable assets for both fundamental research and translational applications in agriculture.

In this study, we elucidate the transcriptional regulation and dynamics of the riboflavin metabolism pathway in Arabidopsis by integrating diverse high-throughput gene expression datasets within developmental and subcellular contexts. The pathway genes exhibit distinct spatial and temporal expression profiles across developmental stages and plant organs. Our analysis further reveals a coordinated transcriptional reprogramming of the riboflavin pathway that maintains flavin homeostasis under osmotic and salt stress conditions, where fluctuations in flavin levels occur. Given the central role of metabolic pathway gene regulation in plant adaptation and metabolic engineering, our findings provide a valuable framework for future biotechnological applications targeting flavin metabolism.

## 2. Methods

### 2.1. Databases and Tools

Genes involved in the riboflavin metabolism pathway in *A. thaliana* were annotated based on Gerdes et al. (2012) [4], the KEGG database [5], and the relevant literature. A complete list of the annotated genes is provided in Supplementary Table S1.

Spatiotemporal expression analysis of the riboflavin pathway genes was conducted using RNA-seq data retrieved from the Transcriptional Variation Analysis database (TraVAdb) [6]. Expression values were normalized (0–1) based on trimmed mean of M-value (TMM) normalization. For each gene in the riboflavin pathway, expression profiles were also obtained using the ATHENA (Arabidopsis thaliana Expression Atlas) platform [7]. ATHENA provides access to quantitative transcriptomic and proteomic data from 30 matched *A. thaliana* (*Col-0*) tissues, comprising more than 25,000 transcripts and 18,000 proteins. Transcript levels are expressed as transcripts per million (TPM), and protein abundance is reported as intensity-based absolute quantification (iBAQ) values. To identify the *cis* elements within the promoter regions (−1500 bp to +100 bp relative to the translation initiation codon) of the riboflavin metabolism pathway genes, the PlantCARE database was utilized [8]. For the expression profile analysis of the Arabidopsis riboflavin pathway genes under abiotic stresses, gene expression values were retrieved from the ePlant database [9] and were used to generate heatmaps based on their normalized expression levels. Heatmaps were generated using TBtools II (v2.326) [10]. To construct the co-expression network of riboflavin pathway genes, co-expression data were retrieved from ATTED-II (version 12.0). For each gene, the top 100 most highly co-expressed genes were extracted and merged into a single dataset. The final network was visualized using Cytoscape (version 3.10.3); nodes with only one connection (degree = 1) were filtered out to improve clarity and interpretability.

### 2.2. Extraction and Quantification of Flavins

*A.thaliana* seeds were surface-sterilized and sown in Petri dishes containing 0.5× Murashige and Skoog (MS) medium (Duchefa, The Netherlands), pH 5.7, supplemented with 1% sucrose and 0.05% 4-morpholineethanesulfonic acid (MES; AppliChem GmbH, Darmstadt, Germany), and solidified with 0.33% phytagel (Sigma, London, UK). Seeds were stratified for 48 h at 4 °C and subsequently grown for 7 days at 22 °C in a Fitotron growth chamber (Weiss Gallenkamp, Loughborough, UK) under long-day conditions (16 h light/8 h dark) with a light intensity of 120 µmol m^−2^ s^−1^. For salt and osmotic stress treatments, seedlings were transferred to the same medium supplemented with 100 mM NaCl and 300 mM mannitol, respectively, and maintained under identical growth conditions. Seedlings were homogenized in flavin extraction solution (methanol:methylene chloride, 9:10 *v*/*v*) at 20 µL mg^−1^ fresh tissue. A 0.1 M ammonium acetate solution, pH 6.0 (9 parts), was added to the extraction solution (19 parts) and vortexed for 2 min. Samples were centrifuged at 16,000× *g* at 4 °C, and flavins in the aqueous phase were analyzed using a Jasco HPLC system (Pump PU-4180, Autosampler AS4050, FP2020 Plus; Jasco, Tokyo, Japan) equipped with a Spherisorb ODS2 column (5 µm, 4.6 × 250 mm; Waters, Milford, MA, USA). The mobile phase consisted of methanol:0.01 N NaH_2_PO_4_ pH 5.5 (3:7 *v*/*v*). A 20 µL sample was injected and eluted at a flow rate of 1 mL min^−1^. Flavins were detected with excitation at 445 nm and emission at 530 nm. Peak identification and quantification were performed using commercially available reference standards of riboflavin (PHR1054, Supelco, Bellefonte, PA, USA), FMN (F8399), and FAD (F6625) purchased from Sigma-Aldrich (St. Louis, MO, USA).

## 3. Results and Discussion

### 3.1. Riboflavin Metabolic Pathway

**Figure 1:**
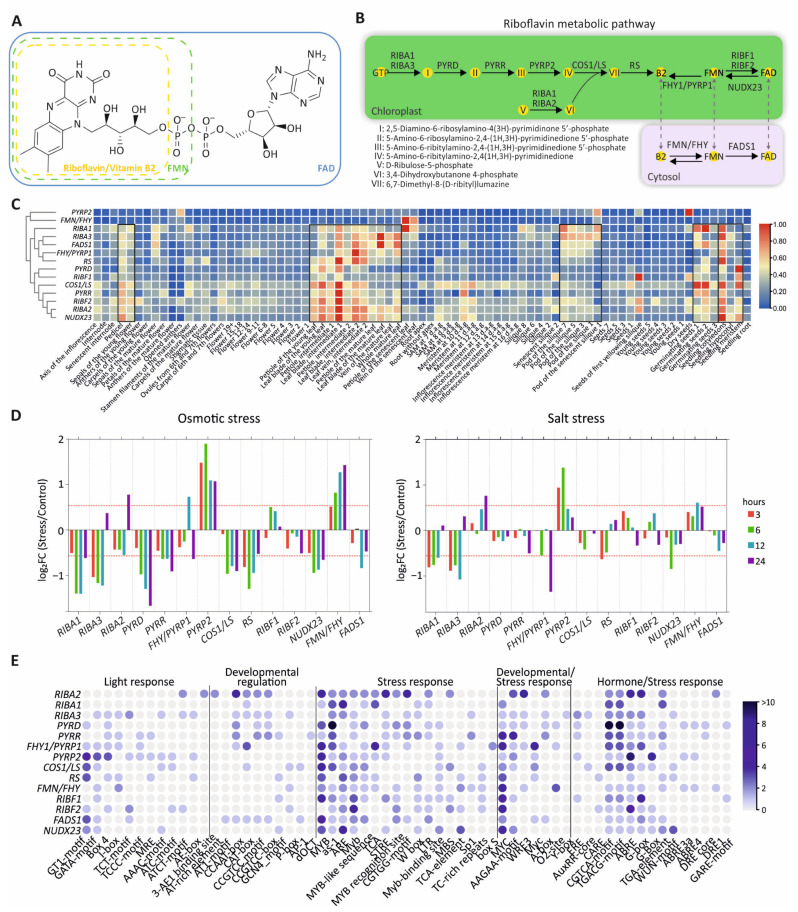
Overview of transcriptional regulation of riboflavin (vitamin B_2_) metabolism in Arabidopsis. (**A**) Chemical structures of riboflavin, FMN, and FAD; the schematic was adapted from Martin et al. 2020 [22]. (**B**) Riboflavin metabolic pathway in *A. thaliana* based on KEGG pathway and Gerdes et al. 2012 [4]. Green and purple panels represent biosynthesis steps located in chloroplast and cytosol, respectively. The shaded arrow indicates flavin transport from the chloroplast to the cytosol, though the underlying mechanism remains unknown. (**C**) Spatiotemporal gene expression analysis of Arabidopsis riboflavin metabolic pathway. RNA-seq data were retrieved from the Transcription Variation Analysis database (TraVADB). Colors denote “0–1”-normalized values of the read counts with the TMM method. Squares indicate tissues and developmental stages with higher gene expression; (**D**) differential expression of flavin-metabolism-related genes under osmotic and salt stress conditions. Bar plots show gene expression fold change (log_2_FC; stress vs. control) of riboflavin metabolism genes at four time points (3, 6, 12, and 24 h) under osmotic stress and salt stress. Positive and negative values indicate upregulation and downregulation of gene expression, respectively, relative to control samples. Red dashed lines denote ±0.5 log_2_FC thresholds. Bar colors correspond to the duration of the stress treatment. Gene expression values were retrieved from the ePlant database [9]; (**E**) distribution of *cis*-regulatory elements within the promoter regions of riboflavin genes. The color scale denotes the number of *cis* elements identified. *Cis*-element analysis was performed using the PlantCARE database [8].

Riboflavin consists of a tricyclic isoalloxazine ring linked to a ribitol side chain, which, upon phosphorylation by riboflavin kinase, forms FMN (Figure 1A). FAD synthetase subsequently converts FMN to FAD by adding an adenosine monophosphate. In plants, riboflavin biosynthesis is initiated from GTP or ribulose-5-phosphate (Ru5P) as precursor molecules and proceeds through seven enzymatic steps within chloroplasts (Figure 1B and Supplementary Table S1). The first step is catalyzed by bifunctional RIBA enzymes, which possess both GTP cyclohydrolase II (GCHII) and 3,4-dihydroxy-2-butanone-4-phosphate synthase (DHBPS) activities [11]. In Arabidopsis, three RIBA isoforms have been identified. RIBA1 retains both enzymatic activities, RIBA2 lacks GCHII activity, and RIBA3 lacks DHBPS activity due to mutations in conserved peptide domains critical for these catalytic functions [11] (Figure 1B and Supplementary Table S1). PYRD, a pyrimidine deaminase, catalyzes the deamination of the pyrimidine ring of 2,5-diamino-6-ribosylamino-4(3H)-pyrimidinone 5′-phosphate, producing 5-amino-6-ribosylamino-2,4(1H,3H)-pyrimidinedione 5′-phosphate. Subsequently, PYRR, which is a pyrimidine reductase, reduces the ribosyl moiety to generate 5-amino-6-ribitylamino-2,4(1H,3H)-pyrimidinedione 5′-phosphate [12]. This intermediate is dephosphorylated in vitro by AtcpFHy/PyrP1 and AtPyrP2 enzymes, with AtPyrP2 shown to be the primary enzyme responsible for this reaction in planta [13]. The next step is catalyzed by lumazine synthase (COS1/LS), which combines DHBP and 5-amino-6-ribitylamino-2,4(1H,3H)-pyrimidinedione into 6,7-dimethyl-8-(D-ribityl) lumazine [14,15]. Riboflavin is then synthesized by riboflavin synthase (RS) and subsequently converted to FMN and FAD [16]. In plants, the bifunctional FMN hydrolase/FHY enzyme, which converts riboflavin to FMN, has been characterized and potentially resides in the cytosol [17] (Figure 1B and Supplementary Table S1). Notably, the FMN/FHY enzyme also catalyzes the dephosphorylation of FMN to riboflavin in vitro, due to FMN hydrolase activity, a function similarly performed by the chloroplast-localized AtcpFHy/PyrP1 enzyme [18]. FAD is synthesized by RIBF1 and RIBF2 in chloroplasts and by FADS1 in the cytosol [19,20]. In addition, NUDX23 exhibits FAD pyrophosphohydrolase activity, hydrolyzing FAD to FMN in chloroplasts [21].

### 3.2. Spatiotemporal Expression of Riboflavin Metabolic Pathway Genes

High-resolution maps, such as the TraVA database [6] and the integrative proteomic–transcriptomic atlas ATHENA [7], provide robust frameworks for system-level functional genomics. To comprehend the spatiotemporal expression of the genes encoding enzymes of the flavin metabolism pathway, we analyzed RNA-seq data from the TraVA database across diverse developmental stages and organs [6] (Figure 1C and Supplementary Table S2). Most genes are highly expressed in leaf developmental stages and organs as well as in pedicels, sepals, seeds, siliques and seedlings, maintaining uniformly low levels of expression in other developmental stages and organs (Figure 1C). These findings are consistent with previous reports showing that riboflavin tends to accumulate in leaves and seeds across various species, including *Lycium chinense*, sweet potato, and green leafy vegetables, where measurements have shown particularly high riboflavin levels in leaves [23,24,25]. In addition, *RIBF1* expression peaked in seeds in the first yellowing silique, whereas *PYRP2* and *FMN/FHY* steadily displayed low expression across all organs and stages and were highly expressed only in dry seeds and senescent leaves, respectively (Figure 1C). Hierarchical clustering showed discrete modules regarding *RIBA1*, *RIBA3*, *FADS*, *FHY/PYRP1,* and *RS* that form a tight cluster, *FMN/FHY* grouped with *PYRP2*, *RIBA2* clustered with *NUDX23*, and *PYRD* associated with *RIBF1* (Figure 1C).

Further, the ATHENA tool, which integrates RNA-seq and mass-spectrometry-based proteomics data from thirty distinct tissues, was employed to simultaneously analyze both the transcriptional and translational profiles of the enzymes involved in the flavin biosynthesis pathway across Arabidopsis tissues. A scatterplot comparing intensity-based absolute quantification (iBAQ) and transcripts per million (TPM) values confirmed a clear enrichment of most enzymes in leaves and seeds, while also demonstrating that these enzymes were detectable across all Arabidopsis tissues (Supplementary Figure S1). This observation is consistent with the TraVA results, further supporting that the majority of flavin biosynthesis genes and enzymes are predominantly expressed and accumulate in leaves and seeds. Hierarchical clustering of proteomics data based on iBAQ values revealed distinct patterns of protein accumulation among the enzymes of the flavin biosynthesis pathway. Notably, RIBA1, RIBA3, FADS1, RIBF2 formed a tight cluster, closely resembling the clustering pattern observed at the transcriptomic level (Supplementary Figure S2). Based on both proteomic and transcriptomic data, an additional cluster was formed by FMN/FHY and PYRP2, whereas the rest of flavin biosynthesis enzymes grouped separately. Taken together, these results support a modular coregulation pattern of flavin biosynthesis genes, highlighting coordinated yet distinct regulatory circuits relative to plant development and organ identity. Most genes show evident stimulation of expression in major metabolic sinks for flavins, including leaves, germinating seeds and seedlings, indicative of high energy demands in these tissues. Interestingly, the stage-specific induction of *PYRP2* in dry seeds and *RIBF1* during silique maturation suggest that riboflavin and FAD supply is fine-tuned by a regulatory checkpoint upon major developmental transitions sustaining reproduction. Notably, FMN/FHY, potentially acting as the key enzymatic hub converting riboflavin to FMN, the active cofactor precursor of FAD, showed consistently low expression across all developmental stages and organs, with a marked upregulation observed only in senescent leaves. This upregulation likely reflects an increased cytosolic demand for FMN and FAD during leaf senescence, where extensive oxidative stress and altered redox homeostasis require enhanced availability of flavin cofactors to support ROS-detoxifying enzymes.

### 3.3. Response of Riboflavin Metabolism Genes Under Osmotic and Salt Stress

Stress conditions modify energy metabolism in plants, relying on flavin cofactor availability, and flavin cofactors play crucial roles in oxidative stress responses and plant adaptation [26]. We therefore investigated the response of riboflavin biosynthesis pathway components to osmotic and salt stress conditions, which are tightly linked to redox balance and metabolic adaptation. Plant eFP Browser integrates large-scale transcriptomic data from RNA-seq and microarrays, enabling the visualization of gene expression across developmental stages, tissues, and stress responses [9]. Relative gene expression, expressed as log_2_ fold change, was analyzed upon osmotic and salt treatments at four time points, namely, 3, 6, 12, and 24 h (Figure 1D and Supplementary Table S3). Strikingly, under both stresses a similar overall expression pattern was observed. Under osmotic stress, a distinct transcriptional reprogramming of the riboflavin metabolism pathway was evident. Most upstream biosynthesis genes encoding enzymes responsible for riboflavin synthesis, including *RIBA1*, *RIBA3*, *PYRD*, *PYRR*, *COS1/LS*, and *RS*, were consistently downregulated across all time points (Figure 1D). This transcriptional response is indicative of a general suppression of riboflavin de novo biosynthesis during osmotic stress at 24 h. In addition, *RIBA2* and *FHY/PYRP1* were upregulated at 24 and 12 h, respectively. Based on the expression profile, the genes involved in flavin metabolism were categorized in two groups. The first group of *NUDX23* and *FADS1* was constantly repressed. *PYRP2* and *FMN/FHY* showed significant induction (Figure 1D). However, *RIBF1* and *RIBF2* displayed minor expression changes. This transcriptional pattern, characterized by FMN/FHY and FHY/PYRP1 induction, accompanied by NUDX23 and FADS1 downregulation, suggests a compensatory mechanism that enhances flavin homeostasis among riboflavin and FMN in the chloroplasts and cytosol under osmotic stress.

Arabidopsis seedlings subjected to salt stress showed an overall similar expression profile, although the transcriptional responses were generally more moderate than those observed under osmotic stress conditions (Figure 1D). In particular, *RIBA1*, *RIBA3*, and *RS* riboflavin biosynthesis genes were clearly suppressed, while *PYRD*, *PYRR*, and *COS1/LS* exhibited minor changes. In contrast, only *RIBA2* among the initial biosynthesis genes was induced. The downstream genes *PYRP2* and *FMN/FHY* were again upregulated, albeit to a lesser extent than under osmotic stress conditions, suggesting that salinity partially affects flavin metabolism. Notably, *FHY/PYRP1* was downregulated after 24 h, mirroring the response observed under osmotic stress. Consistent with our results, previous studies have shown that certain genes associated with the riboflavin metabolic pathway exhibit significant differential expression between salt-tolerant and salt-sensitive plants. Moreover, comparative transcriptomic analyses of salt-sensitive cultivars have further highlighted the involvement of riboflavin metabolism during salinity stress [27].

Taken together, these results indicate that specific isoforms within the riboflavin pathway respond to osmotic and salt stress, likely reflecting specialized roles in cellular adaptation and metabolic reprogramming. In particular, the *FMN/FHY* gene encoding an enzyme responsible for riboflavin conversion into FMN is activated under stress conditions, potentially to support an increased demand for flavin-dependent oxidoreductases in cytosol.

### 3.4. Cis-Element Analysis of Riboflavin Pathway Gene Promoters

These observations of the spatiotemporal transcriptional profile and stress-related responses were further confirmed by analyzing the *cis*-regulatory elements that determine transcriptional regulation of riboflavin biosynthesis genes (Figure 1E and Supplementary Table S4). These *cis* elements were grouped into the categories: “Light response,” “Developmental regulation,” “Stress response,” “Developmental/Stress response,” and “Hormone/Stress response”. Remarkably, several hormone-responsive elements, particularly those associated with ABA and salicylic acid, were identified, suggesting a hormonal control of gene expression under stress conditions. ABRE and G-box *cis*-regulatory elements, which mediate the ABA response [28], and as-1 [29], which is an oxidative stress-responsive element activated by salicylic acid, were highly abundant. Interestingly, ABA activates *PYRD*, *PYRP2*, *COS1/LS,* and *RS* transcription, suggesting a significant role of ABA in flavin metabolism [30]. In addition, multiple stress-responsive elements such as DRE1 (dehydration-responsive element) [31] were highly enriched in *RIBA2* and *FMN/FHY* promoters, further supporting their role in stress adaptation, consistent with their expression response under abiotic conditions (Figure 1E). Promoters of *PYRP2* and *FMN/FHY* genes harbored very few elements related to developmental regulation, consistent with an almost ubiquitous expression across developmental stages and tissues, as previously shown by TraVA expression analysis (Figure 1A).

### 3.5. Co-Expression Hubs of Riboflavin Metabolism Pathway Genes

**Figure 2:**
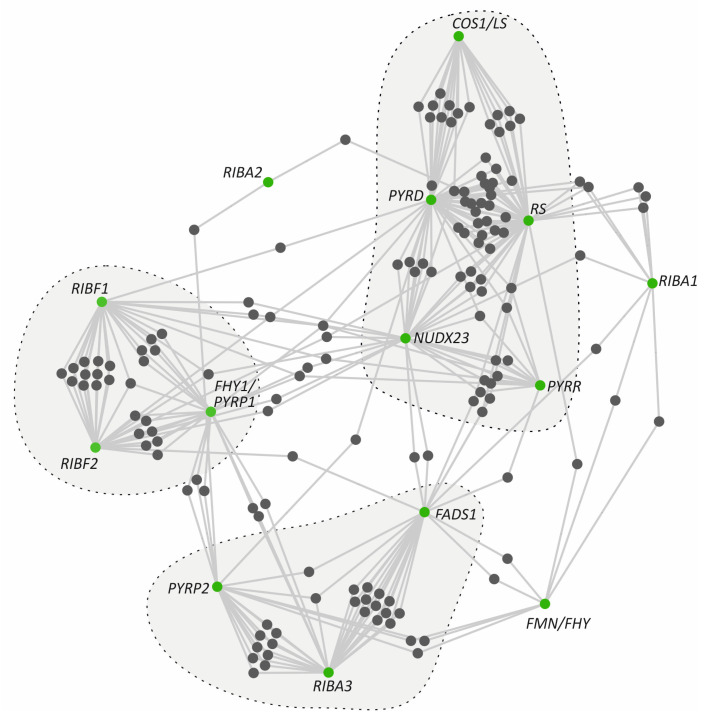
Co-expression network of riboflavin metabolic pathway genes constructed by Cytoscape using co-expression data from ATTED-II. The outlined regions highlight gene clusters exhibiting a tight co-expression pattern, suggesting functional connectivity and coregulation.

Gene co-expression networks enable the investigation of the expression patterns among multiple gene sets under varying conditions. These networks represent the degree of correlation among gene expression profiles within biological samples originating from distinct tissues, developmental stages, and responses to environmental stress conditions [32]. To complement the promoter-level regulatory insights, we next investigated whether the genes of the riboflavin metabolism pathway group in co-expression hubs using the ATTED-II database [33] (Figure 2 and Supplementary Table S5). Interestingly, the initial steps of the pathway driven by *RIBA1* and *RIBA2* did not exhibit any strong co-expression pattern. Nevertheless, *RIBA3*, *PYRP2,* and *FADS1* formed a minor co-expression module, whereas *FMN/FHY* co-expressed with only a limited number of genes, including *RIBA1, FADS1,* and *PYRP2*. Likewise, *COS1/LS*, *PYRD*, *RS,* and *NUDX23* form a tighter cluster, similar to the cluster of *RIBF1*, *RIBF2*, and *FHY1/PYRP1*. Furthermore, *NUDX23* seems to serve as a link among all clusters through its co-expression with multiple genes. Together, these findings support a sub-functionalization model in which flavin metabolism is finely regulated in response to developmental cues and environmental signals, consistent with the distinct expression profiles observed across developmental stages, tissues, and abiotic stress conditions.

### 3.6. Flavin Dynamics Under Osmotic and Salt Stress Conditions

**Figure 3:**
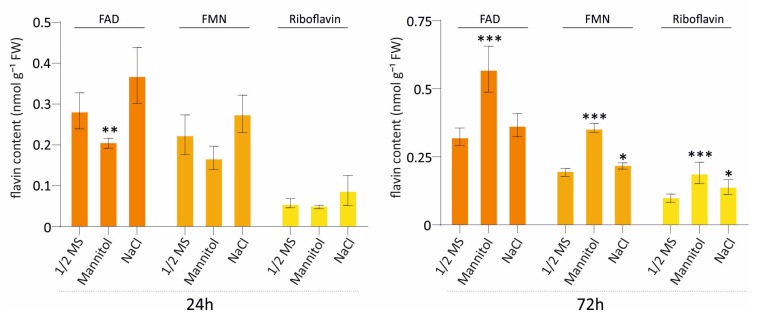
Flavin content in *A. thaliana* seedlings under osmotic and salt stress at 24 h and 72 h. Quantification of FAD, FMN, and riboflavin in seedlings grown on ½ MS medium (control), 200 mM mannitol (osmotic stress), or 100 mM NaCl (salt stress) for 24 h (left panel) or 72 h (right panel). Flavin levels are shown as nmol g^−1^ fresh weight (FW). Bars represent means ± SD (n = 4). Statistical significance relative to the ½ MS control was determined by a two-tailed Student’s *t*-test (*p* < 0.1 = *, *p* < 0.05 = **, *p* < 0.001 = ***).

Based on the transcriptomic differences in riboflavin biosynthesis, we examined flavin levels (FAD, FMN, and riboflavin) following exposure to osmotic stress (mannitol) and salt stress (NaCl) at two time points, 24 h and 72 h (Figure 3 and Supplementary Figure S3). At 24 h, FAD levels decreased under osmotic stress, while a slight, non-significant increase was detected in NaCl-treated plants. By 72 h, these responses were more evident. Under osmotic stress, FAD strongly accumulated, showing a significant increase, while NaCl treatment caused a moderate elevation. FMN also increased under mannitol but to a lesser extent, under salt stress. Riboflavin levels increased in both stress conditions at 72 h, with mannitol-treated samples showing the strongest accumulation profile. Total flavin levels increased exclusively after 72 h under both stress conditions, while no significant changes observed at 24 h (Supplementary Figure S3). Overall, these results show that both osmotic and salt stress affect flavin metabolism, with the most profound differences observed after prolonged stress exposure (72 h).

In Arabidopsis, *AtDREB2G*, a member of *DREB*-family transcription factors, was identified as a positive regulator of flavin biosynthesis under cold stress and abscisic acid (ABA) treatment [30]. In agreement with our results, exposure to stress conditions such as low temperature or ABA application increased riboflavin, FMN, and FAD levels in wild-type Arabidopsis leaves, whereas this response was significantly diminished in *dreb2g* mutants [30]. In this context, cold and ABA induce flavin accumulation via AtDREB2G-mediated transcriptional control. Similarly, salt-treated cotton plants showed increased levels of FMN and FAD in leaves [34]. Furthermore, continuous light, a high oxidative stress condition, was shown to increase riboflavin and FAD levels, but not FMN, in Arabidopsis. This increase was accompanied by induction of several riboflavin-metabolism genes, including *FADS*, *RIBF1*, *RIBF2*, *FMN/FHY*, *COS1/LS*, and *RIBA1* [19]. Under nutrient stress, legume crops also show an increase in flavin levels. In *Medicago truncatula*, iron (Fe) deficiency, particularly when combined with alkaline pH, induced substantial flavin accumulation. Iron-deficient *Medicago* roots accumulated higher total flavin levels (riboflavin, FMN, and FAD) than Fe-deficient plants grown under non-alkaline conditions [35,36]. Similarly, cucumber and melon roots upregulated riboflavin biosynthesis pathway genes under Fe starvation and actively secreted flavin compounds into the rhizosphere. Under low-Fe conditions in cucumber and melon, most riboflavin biosynthesis genes are strongly induced [37].

Our results reveal a general downregulation of the core riboflavin-biosynthesis genes after 24 h of osmotic and salt stress, albeit the effect is more pronounced under osmotic stress. However, the *FMN/FHY* gene, encoding the enzyme that regulates the cytosolic balance between riboflavin and FMN, as well as *FHY1/*PYRP, encoding the enzyme that reconverts FMN to riboflavin in chloroplasts, were both upregulated under osmotic stress conditions. When we analyzed the flavins after 24 h of stress, we detected a decrease in the pathway’s final product, FMN, only under mannitol stress, where we also observed the strongest expression changes. However, clear increases in riboflavin, FMN, and FAD under both stress conditions were evident after 72 h, consistent with previous reports.

Flavin homeostasis under abiotic stress appears to be highly complex. Stress may increase cellular demand for flavins and simultaneously induce enzymes involved in flavin metabolism. In addition, subcellular redistribution of metabolites may occur. For instance, FAD levels may decrease in specific organelles (mitochondria or plastids) due to degradation, while the pool of cytosolic flavins remains constantly buffered. These profiles could also be affected by feedback inhibition mechanisms. In prokaryotes, FMN biosynthesis is controlled by multiple regulatory mechanisms that vary among species. These mechanisms involve riboflavin kinase inhibition by riboflavin itself, redox-dependent modulation of the substrate, or transient subunit assemblies that influence catalysis [38,39,40]. In humans, reaction products (FMN and ADP) can inhibit riboflavin kinase activity, preventing excessive FAD production and limiting FMN conversion to FAD [40]. Additionally, because flavin-dependent proteins are prone to oxidative damage and degradation under stress conditions, their flavin cofactors could be rapidly released and damaged or catabolized [41]. Cells may also actively preserve flavin pools to sustain essential redox reactions, while they simultaneously upregulate components of the flavin-biosynthesis machinery at the enzymatic level. In our case, although a direct link between transcriptional regulation and these regulatory processes was not confirmed, we observed induction of *FMN/FHY* and *PYRP1* 24 h after stress under both osmotic and salt stress, while most genes were downregulated, which may reflect a compensatory response to regulate the abundance of enzymes involved in flavin metabolism and maintaining flavin homeostasis during abiotic stress.

## 4. Conclusions

**Figure 4:**
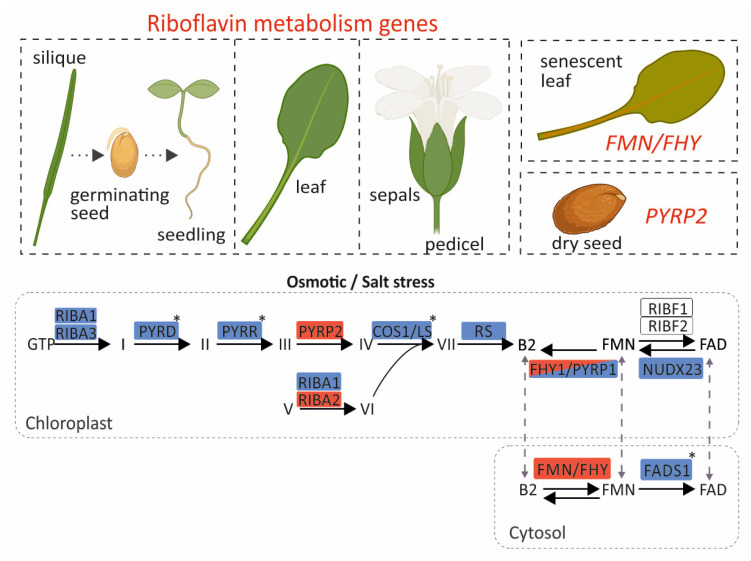
Graphical representation of the transcriptional profile of riboflavin pathway genes in *A. thaliana* across developmental stages, tissues, and abiotic stress conditions. Upregulated and downregulated genes are shown in red and blue, respectively. Genes highlighted in both colors exhibit induction or suppression depending on the stress type. Genes without color show a stable expression pattern. The asterisks mars genes whose expression is altered only under osmotic stress. Most genes display elevated expression in siliques, germinating seeds, seedlings, and leaves. In contrast, *FMN/FHY* and *PYRP2* maintain relatively stable expression across stages and tissues, being induced only in senescent leaves and dry seeds, respectively. This image was created with BioRender (Daras, G. 2025; https://BioRender.com/3p22o59, accessed on 20 August 2025).

Our results reveal that mostly all genes are highly expressed in the pedicel, sepals, leaves, germinating seeds, and seedlings (Figure 4). This is consistent with an elevated metabolic demand for flavins to sustain photosynthesis and development in these tissues, where numerous flavoproteins participate in redox reactions and energy metabolism. Moreover, *RIBA1*, *RIBA3*, *PYRD*, *PYRR*, *COS1/LS,* and *RS,* which participate in riboflavin biosynthesis, were transcriptionally repressed at 24 h under osmotic stress conditions. This transcriptional response potentially reduces the accumulation of intermediate compounds that could otherwise be detrimental to plastids under stress conditions. On the contrary, increases in the expressions of *PYRP2* and *RIBA2* were observed, which could not be justified based on the trends in most genes within the pathway but could instead be attributed to additional moonlighting functions [42]. Such cases have been reported where enzymes of the pathway participate in additional roles beyond riboflavin metabolism. A characteristic example is NUDX23, which, apart from its FAD pyrophosphohydrolase activity—hydrolyzing FAD to FMN—also participates in the post-translational regulation of PSY (phytoene synthase) and GGPPS (geranylgeranyl diphosphate synthase) for carotenoid biosynthesis through direct interactions with PSY and GGPPS [43]. For flavin metabolism, *FHY/PYRP1* and *FMN/FHY* were upregulated under osmotic stress, *FADS1* and *NUDX23* were downregulated, and *RIBF1/2* genes showed a constant expression pattern (Figure 4). Flavin measurements following osmotic and salt stress showed alterations, which were pronounced after 72 h under prolonged stress, and this likely explains the increased expressions of *FHY/PYRP1* and *FMN/FHY1*. This dynamic response seems to regulate FMN homeostasis in the chloroplast and cytosol. This reconfiguration of expression reveals an essential role of riboflavin and FAD in flavoprotein-mediated detoxification and signaling. Meanwhile, it also highlights FMN as a crucial intermediate that preserves a balanced riboflavin/FAD ratio in response to cellular demand. This stress-induced metabolic reprogramming is further supported by promoter analysis, which revealed strong enrichment in *cis*-regulatory elements associated with abiotic stress responsiveness. Co-expression network analysis identified a distinct cluster composed of core riboflavin pathway genes, including *PYRR*, *PYRD*, and *RS*. Interestingly, *RIBA1/2/3*, *FMN/FHY*, and *FADS1,* which encode the initial and terminal enzymes of the pathway, share only a limited number of co-expression partners, suggesting a modular regulatory strategy and distinct functional roles. Within this architecture, *FMN/FHY* emerges as a central node. FMN/FHY catalyzes the conversion of riboflavin into FMN, the direct precursor required for FAD synthesis. It exhibits a unique expression pattern that is generally low but stable, becoming highly stress-responsive and strongly induced in senescent leaves.

By systematically combining large-scale expression profiles, co-expression network analysis, cis-regulatory element enrichment, and flavin metabolite measurements, we uncover previously unknown coordinated and stress-responsive regulatory modules within the riboflavin biosynthesis and interconversion pathways. In particular, we identify differential transcriptional responses among pathway components under osmotic stress. The role of FMN/FHY as a central regulatory node is revealed, linking riboflavin and FMN homeostasis. Furthermore, we provide evidence of the modular regulation of the expression of specific enzymes. Altogether, these findings outline a coordinated transcriptional and regulatory program in the riboflavin pathway that enables plants to maintain flavin homeostasis under stress. Understanding this regulatory network could provide valuable opportunities for genetic engineering of flavin biosynthesis in plants, with promising applications in crop biofortification, stress resilience, and metabolic engineering.

## Data Availability

Processed data are contained within the article and the Supplementary Material. RNA-seq data were retrieved from the Transcriptional Variation Analysis database (TraVAdb) at https://travadb.org/. Expression profiles were obtained using the ATHENA (*Arabidopsis thaliana* Expression Atlas) platform at https://athena.proteomics.wzw.tum.de/master_arabidopsisshiny/ (accessed on 30 August 2025). Cis-element data are available from the PlantCARE database at https://bioinformatics.psb.ugent.be/webtools/plantcare/html/ (accessed on 30 August 2025). Gene expression profiles under abiotic conditions are available from the ePlant database at https://bar.utoronto.ca/eplant/ (accessed on 10 July 2025). Co-expression data were retrieved from ATTED-II at https://atted.jp/ (accessed on 3 July 2025).

## Supplementary Materials

The following supporting information can be downloaded at: https://www.mdpi.com/article/10.3390/genes17010016/s1, Supplementary Figure S1: Scatterplot illustrating the relationship between iBAQ (INTENSITY-BASED ABSOLUTE QUANTIFICATION) and TPM (transcripts per million) values for genes in the flavin biosynthesis pathway in *A. thaliana* across various tissue groups [7]. Plot was generated using the ATHENA tool. “Leaf” and “Seed” tissue groups are indicated. Supplementary Figure S2: Heatmap clustering of proteomics data, with correlations estimated using the Pearson correlation coefficient applied to iBAQ values. Heatmap was generated using the ATHENA tool. Supplementary Figure S3. Total flavin content in *A. thaliana* seedlings under osmotic and salt stress at 24 h and 72 h. Quantification of FAD, FMN, and riboflavin in seedlings grown on ½ MS medium (control), 200 mM mannitol (osmotic stress), or 100 mM NaCl (salt stress) for 24 h (left panel) or 72 h (right panel). Flavin levels are expressed as nmol g^−1^ fresh weight (FW). Bars represent means ± SD (n = 4). Statistical significance relative to the ½ MS control was determined by a two-tailed Student’s *t*-test (*p* < 0.1 = *, *p* < 0.001 = ***). Supplementary Table S1. List of experimentally characterized genes encoding enzymes involved in the riboflavin metabolic pathway in *Arabidopsis thaliana.* Supplementary Table S2. Absolute read counts of the riboflavin metabolic pathway genes across plant tissues determined by RNA-seq and retrieved from the TraVADB database. Supplementary Table S3. Expression profile of the riboflavin metabolism pathway genes in response to abiotic stress conditions. Supplementary Table S4. Identified cis-regulatory elements within the promoter regions of riboflavin metabolism pathway genes. Supplementary Table S5. Data retrieved from ATTED-II used to construct the co-expression network.

## Abbreviations

The following abbreviations are used in this manuscript:

ATTED-II	*Arabidopsis thaliana* trans-factor and cis-element prediction database-II
COS1/LS	*coi1* Suppressor1/Lumazine Synthase
FAD	Flavin Adenine Dinucleotide
FMN	Flavin Mononucleotide
FADS1	FAD Synthetase
FMN/FHY	FMN Kinase/FMN Hydrolase
FHY1	FMN Hydrolase 1
iBAQ	Intensity-Based Absolute Quantification
NUDX23	Nudix Hydrolase 23
PYRD	Pyrimidine Deaminase
PYRP	Pyrimidine Phosphatase
PYRR	Pyrimidine Reductase
RIBA	Riboflavin Biosynthesis Protein
RS	Riboflavin Synthase
TMM	Trimmed Mean of M-values
TPM	Transcripts Per Million
TraVAdb	Transcriptome Variation Analysis Database
