# Supplementary figures and images for: Developmental and Stress-Mediated Transcriptional Shifts in Riboflavin Metabolism Pathway in Arabidopsis
Source: Genes (Basel). 2025 Dec 25;17(1):16. doi: 10.3390/genes17010016 (PMC12840815; doi:10.3390/genes17010016)

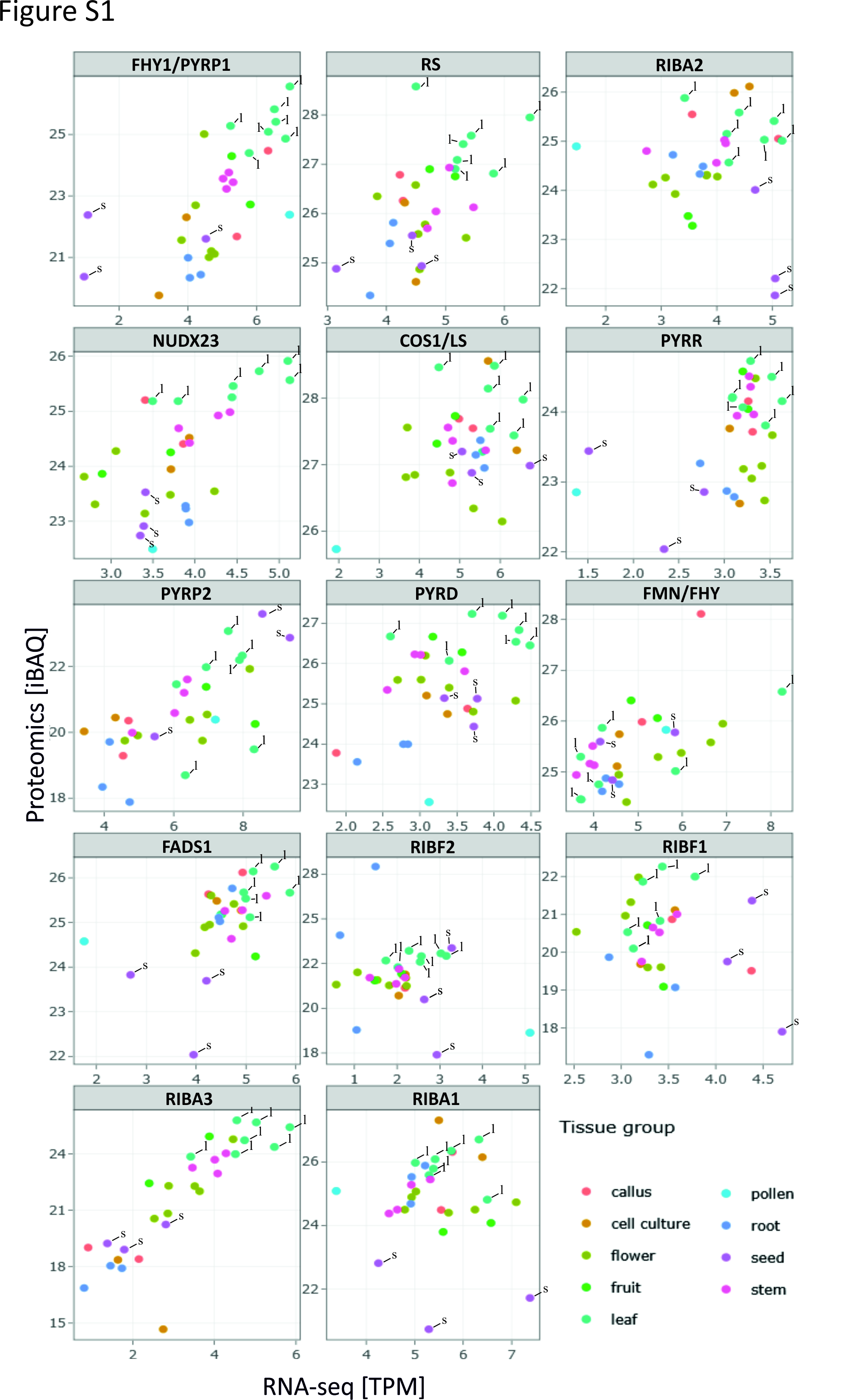

Supplement: Supplementary file 1 [file genes-17-00016-s001.zip › Figure_S1.jpg]

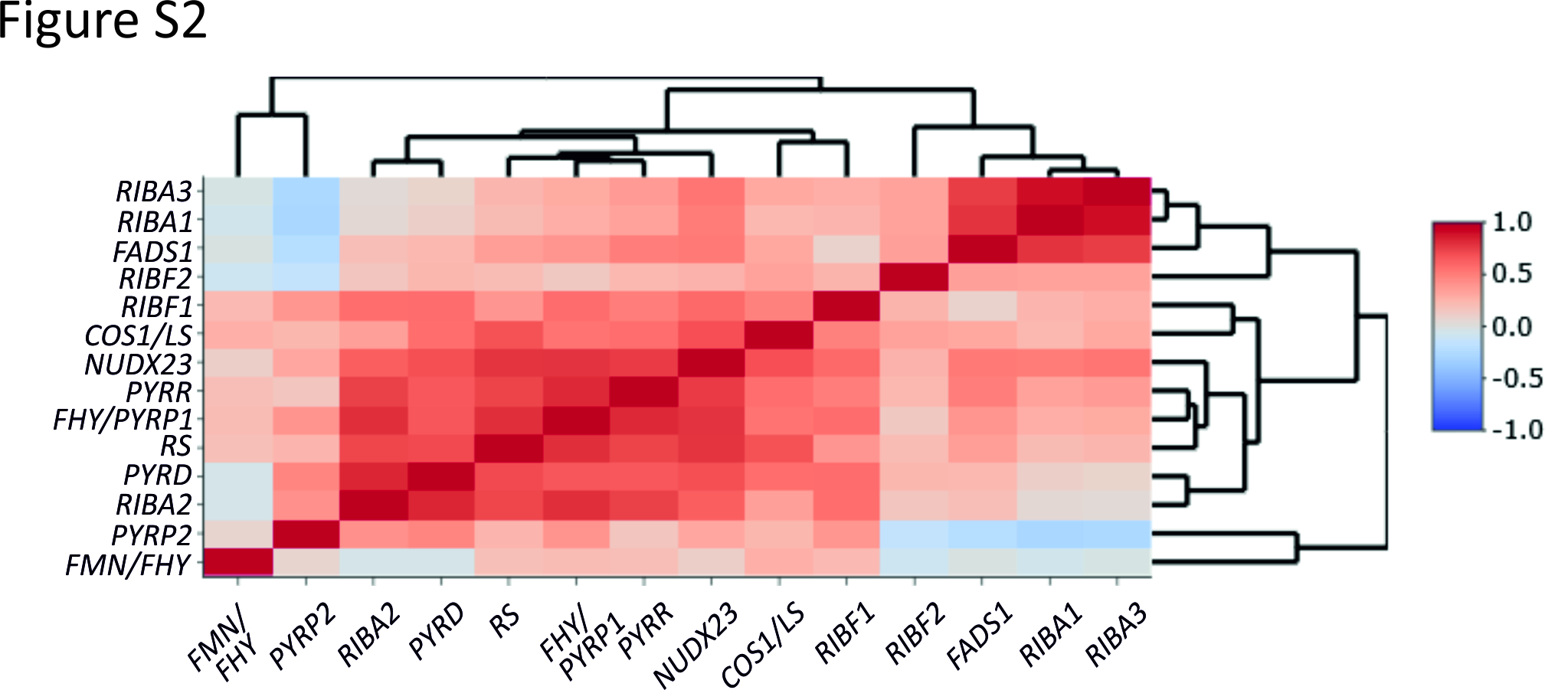

Supplement: Supplementary file 1 [file genes-17-00016-s001.zip › Figure_S2.jpg]

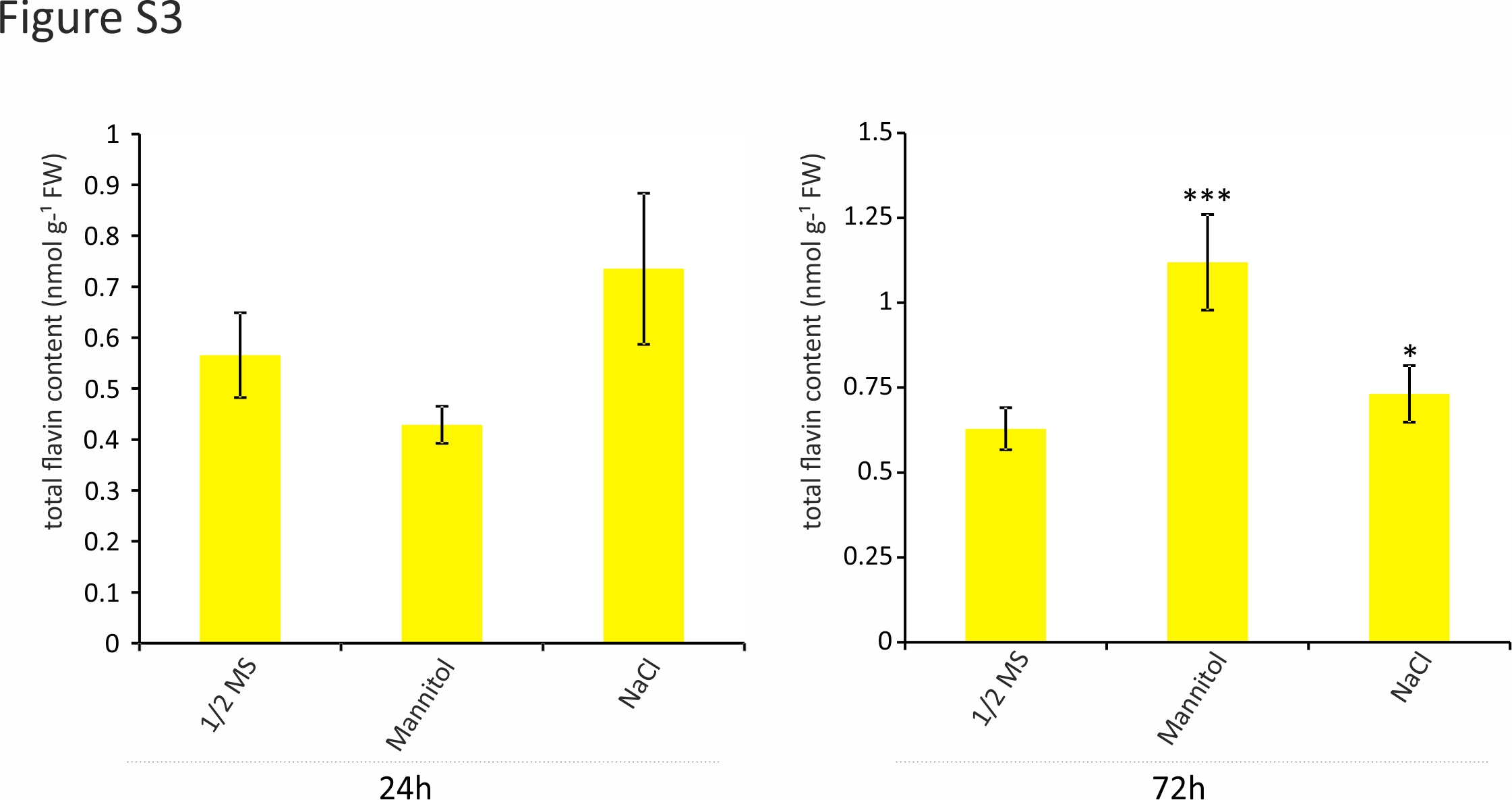

Supplement: Supplementary file 1 [file genes-17-00016-s001.zip › Figure_S3.jpg]
